# The Potential and Hurdles of Targeted Alpha Therapy – Clinical Trials and Beyond

**DOI:** 10.3389/fonc.2013.00324

**Published:** 2014-01-14

**Authors:** Jörgen Elgqvist, Sofia Frost, Jean-Pierre Pouget, Per Albertsson

**Affiliations:** ^1^IRCM, Institut de Recherche en Cancérologie de Montpellier, Montpellier, France; ^2^INSERM, U896, Montpellier, France; ^3^Université Montpellier 1, Montpellier, France; ^4^Institut Régional de Cancérologie de Montpellier, Montpellier, France; ^5^Fred Hutchinson Cancer Research Center, Seattle, WA, USA; ^6^Department of Oncology, University of Gothenburg, Gothenburg, Sweden

**Keywords:** targeted alpha therapy, alpha emitters, radionuclide therapy, dosimetry, ovarian cancer, cancer

## Abstract

This article presents a general discussion on what has been achieved so far and on the possible future developments of targeted alpha (α)-particle therapy (TAT). Clinical applications and potential benefits of TAT are addressed as well as the drawbacks, such as the limited availability of relevant radionuclides. Alpha-particles have a particular advantage in targeted therapy because of their high potency and specificity. These features are due to their densely ionizing track structure and short path length. The most important consequence, and the major difference compared with the more widely used β^−^-particle emitters, is that single targeted cancer cells can be killed by self-irradiation with α-particles. Several clinical trials on TAT have been reported, completed, or are on-going: four using ^213^Bi, two with ^211^At, two with ^225^Ac, and one with ^212^Pb/^212^Bi. Important and conceptual proof-of-principle of the therapeutic advantages of α-particle therapy has come from clinical studies with ^223^Ra-dichloride therapy, showing clear benefits in castration-resistant prostate cancer.

## Introduction

**Figure 1:**
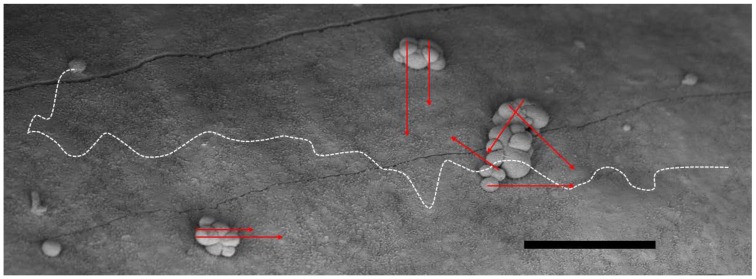
**The favorable geometric situation for α-particles in small-scale metastases (e.g., in the adjuvant setting) is depicted in a scanning electron microscopy micrograph of micro-metastatic clusters from ovarian cancer on the peritoneal lining (mouse)**. The range of the α-particles in red (here ~50–70 μm), can hardly reach the surrounding normal healthy cells other than possibly the mesothelium and its sub-layer. They cannot reach the epithelial cells of the intestinal lining. The situation for β^−^ particles on the other hand, shows that a great deal of its energy will be deposited far away from the binding site and possibly into healthy tissue as demonstrated by the white dashed line (here ~700 μm). Consequently, it may add to side effects. Bar equals 100 μm.

In radioimmunotherapy (RIT), monoclonal antibodies (mAb) are conjugated to radionuclides, which provide a specific internal radiotherapy. The clinical success so far has been achieved with the beta (β^−^)-emitting (electrons) nuclides ^90^Y and ^131^I, conjugated to anti-CD20 mAb in follicular B-cell non-Hodgkin lymphoma. The lack of success in the adjuvant setting in solid cancer (i.e., with microscopic tumor burden) may be due to the fact that emitted electrons do not deposit their main energy to the micro-metastatic tumor cells where the antibody has bound; rather, the energy (and its effects) will be released along a several millimeter long electron track, i.e., in the surrounding healthy tissue, see Figure 1.

This review concerns targeted alpha (α)-particle therapy (TAT), where α-emitting nuclides are conjugated to a carrier, normally an antibody. Alpha-particle decay is the release of a heavy and energetic particle, which deposits its energy in a 70–100 μm long track, i.e., within microscopic tumor cell clusters. Importantly, this high linear energy transfer (high-LET) radiation is not dependent on active cell proliferation or oxygenation, and the resulting DNA damage caused by α-particles is much more difficult to repair than that of β^−^. Thus, highly cytotoxic radiation directed to the relevant tumor cell deposits holds the promise of adding substantially to hitherto failing curative adjuvant chemotherapy both when administered intraperitoneally (i.p.) for ovarian cancer, and as a systemic curative adjuvant treatment for breast, colon, prostate, and other malignancies, constituting a “*systemic conformal radiotherapy at the cellular level*.”

Monoclonal antibodies are so far the most commonly used vector (1, 2). Other targeting agents include substrate analogs, normally in the form of peptides (3, 4), or ligands like folic acid (5). The mAb can be the whole immunoglobulin molecule or fragments like F(ab′)_2_ or single chain, diabodies, etc. Clearance and tumor uptake vary with size and pharmacokinetic properties, and mAb can now even be tailor-made (6).

A brief introduction to the relatively small number of early stage clinical studies using TAT in a variety of situations will follow, i.e., in recurrent brain tumor (7–9), recurrent ovarian cancer (10), human epidermal growth factor receptor-2 (HER-2) positive i.p. cancers (11), myelogenous leukemia (12–16), non-Hodgkin lymphoma (17), and metastatic melanoma (18, 19). There is also one randomized placebo-controlled trial using ^223^Ra-dichloride (having a high affinity for bone tissue) for symptomatic skeletal metastases in prostate cancer, the use of which is now approved by the US Food and Drug Administration (FDA) (20).

## How Could TAT be Integrated in the Clinic?

Today, the multimodal therapeutic approach often includes local gross-tumor eradication by surgery or external radiotherapy, together with or followed by regional adjuvant radiotherapy, and eventually systemic adjuvant chemotherapy. The order of these interventions may differ. As outlined, TAT is mainly aimed at microscopic residual disease and is therefore perhaps best used after adjuvant chemotherapy, but the timing and situation can vary. A number of thematic situations where TAT has, or may, be used are shortly discussed, relating both to the route of administration and/or a specific intention.

*Intra-cavity* administration is a natural starting point for the introduction of TAT in humans. By this approach, the risk of general side effects of critical organs, e.g., bone marrow, is minimized. Similarly, it reduces the risk of unknown toxicity due to unforeseen microscopic accumulation of the radioimmuno-complex elsewhere in the body. This relates to the use of α-particle emitters with relatively short half-life, such as ^213^Bi (~45 min) and ^211^At (~7.2 h), because most of the radioactive decay will occur within the specific cavity before the substance is distributed throughout the body via the systemic and lymphatic systems. Indeed, this has been proved in recurrent malignant gliomas and for i.p. treatment of ovarian cancer (9–11). In tumor resection cavities, the anti-tenascin mAb ^211^At-81C6 was administered to 18 patients with recurrent brain tumors with no grade 3 or higher toxicity, and it was concluded to be a safe treatment with some positive effects (9). With equally low toxicity, the small 11-amino acid peptide substance P (targeting the neurokinin type-1 receptor) conjugated to ^213^Bi has been either injected in residual tumor or in the resection cavity of glioblastoma multiforme (7, 8).

The i.p. route of administration was used in nine patients with recurrent ovarian carcinoma using ^211^At-MX35, an antibody against sodium-dependent phosphate transport protein 2b (NaP_i_2b) (10). The toxicity was mild, grade I–II, and specifically, there was no bone marrow toxicity. This was likely related to the fact that only 6% of injected initial activity concentration of the infused solution could be measured in serum, which peaked at 45 h. Additionally, ^212^Pb conjugated to trastuzumab, an anti-HER-2/neu receptor, for patients with HER-2 positive i.p. cancer has corroborated a low systemic distribution (11).

*Adjuvant treatment* for large tumor groups, e.g., breast, colorectal, and lung cancer, today includes systemically delivered chemotherapy. Although there is a clear effect on survival, in the case of colon cancer, at most, about 30% of patients harboring micrometastases are cured (21). Similarly low, or lower, figures for the total efficacy of adjuvant chemotherapy apply for breast and other adjuvant therapies. It is thought that TAT could be suitable for a boost, or consolidating, therapy after primary surgery and adjuvant chemotherapy. Besides the more common epithelial cancer where adjuvant chemotherapy is used, it has been suggested that malignant melanoma might benefit from adjuvant TAT. ^213^Bi-9.2.27, an antibody against human neural/glial antigen 2 (NG2), has been administered both intra-lesionally and i.v. in patients with metastatic melanoma with promising results (18, 19). The adjuvant situation is also the goal in ovarian cancer, with the benefit of using local i.p. administration (10). In future clinical trials, however, patients who would remain disease-free even without such an adjuvant therapy might be included. It will therefore be important to include stochastic and long-term risk assessments, such as secondary cancers and/or specific organ dysfunctions, in the therapy justification. In these cases, the equivalent absorbed doses in all relevant organs should be calculated, including a conservative estimate of the relative biological effectiveness (RBE) for the emitted α-particles (22).

If tumor dissemination is confined to the peritoneum today, extensive cytoreductive surgery with i.p. chemotherapy is suggested for selected patients, and i.p. TAT may be used as an additional boost therapy. An analogous local adjuvant treatment situation would be after surgery for peritoneal or pleural mesothelioma. Other multiple special-case scenarios include, e.g., optimized treatment of neuroendocrine tumors expressing somatostatin receptors, using the synthetic ligand octreotate (23), which today are treated with β^−^-particles such as ^177^Lu, if kidney toxicity could be shown to be less. In the diffuse-type gastric cancer subset, TAT using, e.g., a mutated E-cadherin mAb may represent an option for treatment (24).

*Palliative treatment* can be envisaged for relief of specific symptoms from localized disease using the intra-cavity route of administration like meningeal, pleural, or peritoneal carcinomatosis; the latter is currently being explored (11). Prolongation of life was found with i.v. injected ^223^Ra-dicloride (Xofigo^®^, formerly named Alpharadin) in a placebo-controlled phase III trial for castration-resistant prostate cancer metastases (20). Although ^223^Ra-dicloride is not conjugated to a targeting molecule, it can be considered as targeted on the basis of its affinity for bone tissue, due to similarities to calcium. The other study objectives, to give symptom relief of bone metastasis and reduce skeletal events, were also fulfilled. Hematological toxicity was surprisingly low and a good tolerability is truly important in palliative treatment. This drug is now also investigated for retreatment (25) and use in combination treatment with docetaxel (26) and also in osteosarcoma (27). A true targeted therapy (i.e., a radionuclide bound to a *tumor*-specific agent) in early stage prostate cancer, with only minimal metastatic disease, could be used before the appearance of bone metastasis-related symptoms. At the time when only the prostate specific antigen (PSA) level has started to increase, after optimal local and endocrine treatment, as a possible adjunct PSA salvage treatment.

**Figure 2:**
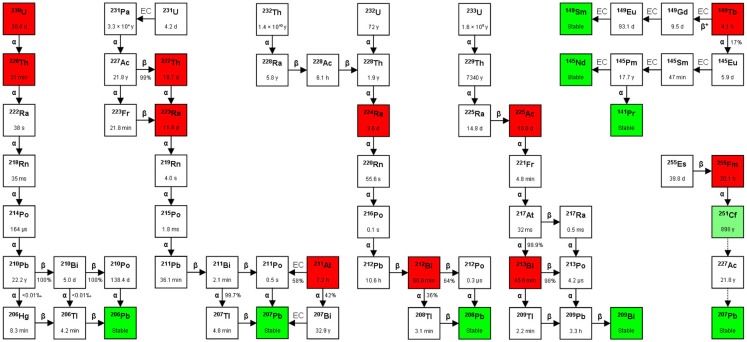
**Decay chains**. Alpha-particle emitters are in red boxes and stable isotopes are in green boxes. The box in light green to the far right (^251^Cf) indicates that although the isotope is considered stable in medical applications (*T*_1/2_ = 898 years), it can still decay via ^227^Ac to ^207^Pb (stable). The *T*_1/2_ is shown inside each box, and between boxes the type of decay [α, β^(−/+)^, or EC (electron capture)], with the probability of each decay route occurring (expressed as %). In the figure are also shown three alpha-particle emitters that are not mentioned in the text: ^230^U, ^226^Th, and ^255^Fm. Studies on the feasibility of producing ^230^U and its daughter ^226^Th via proton irradiation of ^231^Pa according to the ^231^Pa (p, 2n) ^230^U reaction have been performed (29). So far, there are no published data on the use of these three nuclides for TAT, although ^255^Fm has been occasionally mentioned as a potential candidate for targeted radionuclide therapy.

*Systemically dispersed myelo-lymphoproliferative malignancies* are more rapidly accessible for radioconjugate binding compared with solid tumors, when considered as floating cell suspensions. However, they do form extensive aggregates in the bone marrow and in peripheral lymphoid tissues. RIT with the longer range, low energy β^−^-particle-emitting conjugates (Zevalin^®^/Bexxar^®^) is useful for the more bulky lymphomas and are approved for follicular B-cell non-Hodgkin lymphoma, but comes with long-lasting bone marrow toxicity (28). The safety and feasibility of TAT with ^213^Bi-lintuzumab (HuM195), a humanized anti-CD33 mAb that targets myeloid leukemia cells, has been established (12, 14). Importantly, anti-leukemic effects were also demonstrated, providing the first proof-of-concept in human (12). It is suggested that when introducing TAT directly after chemotherapy, the cytoreductive effect of the chemotherapy can enhance the possibility of a saturation of CD33 sites by the targeted drug, which will increase the number of radionuclides delivered to leukemia cells without the need for activity escalation (13). To even further enhance the effects, the same mAb is now being conjugated to the *in vivo* α-particle generator ^225^Ac, which decays in a serie emitting four α-particles (15), see Figure 2. Additionally, an on-going investigation is using the combination of ^225^Ac-lintuzumab and the cytotoxic drug cytarabine in older patients with acute myeloid leukemia (AML) (16). The surface targets used today are mostly present to a certain degree on normal hematological cells. Therefore, bone marrow toxicity is of concern and more malignant cell-specific targets are warranted.

*Regarding manifest macroscopic disease*, as has been argued, this situation might not be theoretically optimal for TAT. However, there are some clinical indications that TAT may actually be of use also for treating macroscopic tumors. Firstly, there is an interesting phase I trial for manifest stage IV malignant melanoma with promising results, including an objective partial response rate of 10 and 40% of patients having stable disease at 8 weeks (19). A total of 38 patients were treated with the 9.2.27 mAb (against human melanoma chondroitin sulfate proteoglycan) conjugated to ^213^Bi. Secondly, preliminary reports of a phase I dose escalation trial with ^213^Bi-labeled anti-CD20 against relapsed or refractory non-Hodgkin lymphoma preliminary showed no acute or extramedullary toxicity in two responders out of nine treated patients (17). These results are even more promising considering the short half-life of ^213^Bi (~45 min), since a more long-lived nuclide would likely have been able to penetrate the tumor masses better, with possibly even better therapeutic effects. Thus, it is argued that if penetration is optimized and high enough activity is delivered to yield homogenous curative doses, also tumors in the size range of 5–10 mm can be eradicated, as has been shown experimentally (30). This potential could even be further enhanced with the use of pre-targeting strategies (see separate section).

### The ovarian cancer example

The ovarian cancer example aims to use RIT as a locally injected adjuvant therapy. Unfortunately, epithelial ovarian cancer (EOC) mortality has not decreased during the last decades, despite a decline in incidence and treatment intensification. Diagnosis is commonly made at an advanced stage with widespread peritoneal dissemination; 70–75% of the patients are diagnosed at more advanced stages i.e., >stage I. Standard therapy for stage II and higher constitutes surgery with cytoreductive intent (i.e., removal of as much as possible of the macroscopic tumors from the peritoneal surface including bilateral salpingo-oophorectomy), supplemented by i.v. chemotherapy, and sometimes i.p. chemotherapy (31). To enhance survival, trials have assessed the use of whole abdominal or moving-strip external-beam radiotherapy (EBRT) (32), or non-specific i.p. radiotherapy with colloid preparations of ^198^Au or ^32^P as adjuvant therapies (33, 34). However, the results of these studies have not justified their routine use and long-term toxicity in normal tissues is a major concern. However, even when cytoreductive surgery and chemotherapy result in complete remission at second-look laparotomy and normalization of the serum marker cancer antigen 125 (CA-125), about 70% of patients with stage III ovarian cancer will relapse. Recurrence is often characterized by gradual development of ascites and chemotherapy-resistant tumor cells, growing as peritoneal microscopic cell deposits, eventually leading to intestinal adhesions and bowel obstruction.

Chemotherapy injected i.p. in the abdominal cavity can result in both a reduction in recurrences and a decrease in mortality, although at the cost of increased normal tissue toxicity (35, 36). The advantage of i.p. administration compared with i.v. injection for localizing radiolabeled mAb to microscopic peritoneal tumor disease was shown in earlier studies, both in animal models and in patients (37, 38). Therefore, local treatment with the β^−^-particle-emitting radioconjugate ^90^Y-HFMG (human milk fat globule-1, a mAb toward MUC-1) was investigated in a large randomized controlled phase III trial, but overall survival did not improve, although a slight decrease in local intraperitoneal recurrence was observed (39, 40). This negative result might be in part explained by the delivery of a too low absorbed dose from the emitted β^−^-particles to single tumor cells or micrometastases. Consequently, i.p. TAT using specific mAb labeled with α-particle-emitting radionuclides, with the higher LET and shorter path length than β^−^-particles, could be more effective. A phase I study has used the mAb MX35 F(ab′)_2_ fragments labeled with ^211^At, that was administered as i.p. infusion to patients with relapsed ovarian cancer but after having achieved a complete macroscopic response on second-line chemotherapy (10). The tolerability was very good and it was concluded that this treatment could achieve therapeutic absorbed doses in microscopic tumor nodules without causing any radiation-related toxicity (10).

## Radionuclides

Some important physical characteristics of relevant α-particle emitters are presented below, with reference to studies on their therapeutic applications. See Figure 2 for a schematic of the different decay pathways. Importantly, as it is not possible to directly measure the α decay *in vivo*, even a small amount of accompanying γ-radiation will enable scintigraphic evaluation for pharmacokinetic and dosimetric studies to be performed. All α-particle emitters with a serial decay that includes α-particle daughters can present problems, as the daughters will detach from the targeting vector due to the elevated recoil energy (up to 200 keV). Such free nuclides can then diffuse away, leading to untargeted irradiation of normal tissues. Using microdosimetry, the energy deposited in the target could be reduced by 50%, as has been calculated for the ^211^At α-particle-emitting daughter ^210^Po, with a *T*_1/2_ of 0.5 s (41).

*Actinium-225* (*^225^Ac*) has a *T*_1/2_ of 10 days, causing the emission of four α-particles in a serial decay. The decay is accompanied by γ-radiation. This nuclide can have great therapeutic potential when radiochemistry can produce stable binding to ^225^Ac and its daughters. This nuclide is available as a consequence of producing ^233^U via the nuclear reaction ^232^Th (n, γ) ^233^Th (β^−^) ^233^Pa (β^−^) ^233^U for nuclear energy and nuclear weapons purposes decades ago (Figure 2). The possibility of producing ^225^Ac by use of a cyclotron via the ^226^Ra (p, 2n) ^225^Ac is now also investigated (42). ^225^Ac is currently tested in two clinical studies where it is conjugated to the anti-CD33 mAb HuM195 (15, 16).

*Radium-223* (*^223^Ra*) has a *T*_1/2_ of 11.4 days and emits four α- and two β^−^-particles in the decay chain as well as γ-rays, until the stable isotope ^207^Pb is obtained. This nuclide can be produced by neutron activation of ^226^Ra by the nuclear reaction ^226^Ra (n, γ) ^227^Ra (β^−^) ^227^Ac (Figure 2). ^223^Ra is an alkaline earth metal ion and similarly to calcium ions, it accumulates in the bone. To this aim, ^223^Ra-dichloride was developed and is now FDA-approved for bone metastases in castration-resistant prostate cancer (20).

*Bismuth-213* (*^213^Bi*) decays with a *T*_1/2_ of 45.6 min to ^209^Bi (stable), during which it emits one α-particle and an accompanied 440 keV γ-radiation. This nuclide can be obtained by elution of the ^225^Ac/^213^Bi generator, thereby making availability and dispersion to clinical centers possible. The generator is produced by the Oak Ridge National Laboratory in the USA and by the Institute for Transuranium Elements in Karlsruhe, Europe. Although the drawback of its short half-time puts high demand on the logistics for radiochemistry and treatment, ^213^Bi has still been the most used TAT nuclide in clinical trials so far (12–14, 17–19).

*Bismuth-212* (*^212^Bi*) has a *T*_1/2_ of 60.6 min and emits one α- and one β^−^-particle. High energy (2.6 MeV) γ-rays are emitted in the decay; therefore, patients must be treated using special radiation protection routines. This nuclide is available as a consequence of producing ^233^U via the nuclear reaction ^232^Th (n,γ) ^233^Th (β^−^) ^233^Pa (β^−^) ^233^U (n,2n) ^232^U for nuclear energy and nuclear weapon purposes decades ago (Figure 2). The last step in which ^232^U was produced via the (n, 2n) reaction was an unwanted side reaction during the production of ^233^U (Figure 2). However, the parent nuclide of ^212^Bi is the β^−^-emitter ^212^Pb, having a *T*_1/2_ of 10.6 h. The chelator TCMC is used with ^212^Pb and functions as an *in vivo* nanogenerator for the α-particle emitter ^212^Bi. The University of Alabama (USA) has started a clinical trial to evaluate ^212^Pb-TCMC-trastuzumab toxicity levels and anti-tumor efficacy in patients with HER-2 positive cancers in the abdominal cavity (11).

*Astatine-211* (*^211^At*) decays with a *T*_1/2_ of 7.2 h and emits an α-particle in both of the two possible decay routs to the stable nuclide ^207^Bi. Scintigraphy and standard dosimetry are possible due to the accompanying γ-radiation. The limited availability is currently a main obstacle for a wider use of this nuclide, as it can only be cyclotron produced (43). It has been used in clinical trials, locally administered in surgical resection cavities and i.p. as previously discussed (9, 10).

## Dosimetry

Dosimetry was originally developed for radiation protection (44) and diagnostic imaging (45), but is now also needed for optimization of the therapeutic situation using radiopharmaceuticals. The basic concepts of dosimetry are presented in two Medical Internal Radiation Dose (MIRD) publications (46, 47).

α*-Particle dosimetry* takes into account a number of different parameters, particularly the short path length of α-particles in tissue (~100 μm) and the inhomogeneous distribution of α-radiopharmaceuticals in tumors and tissues. Thus, predicting the biological effect based on mean absorbed dose in a tumor or organ might be misleading in some circumstances. The high-LET (~100 keV/μm) and varying LET (with a maximum at the Bragg peak) along the α-particle track are also parameters that have to be taken into account when performing α-particle dosimetry.

The RBE of α-particles ranges from 3 to 7, i.e., α-particle irradiation is 3–7 times more therapeutically effective, or toxic, per unit of absorbed dose than photons or electrons (47). In TAT clinical studies, an RBE of five has been applied to estimate the equivalent absorbed doses (10, 14, 48). The weighting factor applied when estimating the effective (or equivalent) absorbed dose (expressed in Sv, Sievert) is related to the stochastic effects of radiation, e.g., cancer induction. A factor of 20 is commonly recommended for the stochastic effects of α-particles that should however *not* be used when predicting the therapeutic efficacy or toxicity in patients who receive TAT treatment. Indeed, this weighting factor was conservatively derived for radiation protection and was never meant for estimating the deterministic effects relevant to therapy (47). Also, the clinical experience with α-particles is sparse, and therefore the tolerance to absorbed doses in humans has yet to be determined.

α*-Particle dosimetry in the clinic* require pharmacokinetic data similar to those that are required for conventional β^–^-particle therapies (22), e.g., urine, blood, and peritoneal fluids in the case of i.p. treatment (10). All α-particle emitters used so far in clinical studies (^211^At, ^213^Bi, ^223^Ra, ^212^Bi, and ^225^Ac) emit γ-photons, characteristic X-ray, or bremsstrahlung radiation. Using the γ-camera makes quantification of biodistribution possible. The spatial resolution of such images is, however, fairly low. Also, the injected activity is much lower than in a diagnostic setting, generally resulting in a poor signal-to-noise ratio. For similar reasons, 3-Dimensional single-photon emission computerized tomography (SPECT) imaging of the activity distribution in patients is time-consuming. The accuracy could be increased using co-registration techniques with computed tomography (CT) images (49).

Obviously, the absorbed dose in tumors and normal tissues need to be estimated from preclinical studies before initiating treatment studies. However, clinical quantification with the γ-camera can only give an estimate of the uptake of the radiopharmaceutical in whole organs and in macroscopic tumors, while quantification of the absorbed dose in smaller compartments in organs or microscopic tumors is hardly achievable. In TAT, the targeted tumors are often too small to be detected and, at best, indirect methods can be used for estimating the absorbed dose.

With regard to normal tissue protection, in certain cases, blocking agents can be used. For example, both astatine and iodine belong to the halogen elements and pre-treatment with potassium perchlorate can effectively prevent uptake of free ^211^At in cells expressing the sodium-iodine symporter (NIS), e.g., in the thyroid (10).

In the case of i.p. TAT for ovarian cancer, a control γ-camera image of the abdominal region with a radioactive-tracer analog to assure free distribution of the fluids is important. The radioactive flow out of the abdominal cavity can also be determined using a radioactive-tracer analog, by monitoring the activity concentration in blood over time (10). Pharmacokinetic data show that the variation in the absorbed dose in bone marrow can be around 20% (10). If the bone marrow is the dose-limiting organ, its absorbed dose then determines the maximal tolerated activity (MTA), and a radioactive-tracer analog study will be crucial for estimating the patient-specific MTA. However, for i.p. TAT, no effect on the hematopoiesis was recorded (10). Instead, other organs might determine the MTA, possibly the peritoneum; therefore, the activity concentration in the peritoneal fluid is crucial to calculate.

α*-Particle dosimetry on the cell level* should be used when macrodosimetry cannot explain the results of an experiment or when it adds value to the macrodosimetric method (50). For α-particles, the biological effect of just a single ionization event could be so large that the calculation of the mean absorbed dose in a tumor as a whole can be very misleading.

Hence, there is a need for microdosimetry when the statistical variation of the deposited radiation is not minimal in the target such as a cancer cell nucleus. The conceptual framework of microdosimetry that takes into account the stochastic nature of energy deposits in small microscopic targets was proposed almost 60 years ago (51), and the International Commission on Radiation Units and Measurements (ICRU) report No. 36 from 1983 defined all the microdosimetric concepts. Calculations and experiments have shown that as few as five high-LET α-particle traversals through the cell nucleus are enough to kill a cell, whereas 10,000–20,000 low-LET β^–^-particles are needed to achieve the same biological effect (52–54).

Importantly, microdosimetry should be considered for non-targeted but critical tissues, even if it receives a very low mean absorbed dose (47).

## The Biology in Targeted α Therapy

The way high-LET radiation like α-particles interact with biological matter has been described earlier (53, 55–60). They produce dense ionizations along a linear track and generate locally multiple damage sites in sensitive targets like DNA. These lesions, produced in close proximity to each other, are poorly repairable, thus making α-particles highly deleterious (61, 62). While conventional EBRT is characterized by high absorbed doses delivered in a very short time in a homogenous way, TAT and radionuclide therapy in general are characterized by a low absorbed dose rate, protracted exposure, and heterogeneous energy deposit (63).

In EBRT, physical events predominate in the final outcome of the therapy, and most of the effects can be correlated to the absorbed dose according to a linear, linear-quadratic, or sigmoid relationship. Conversely, physical characteristics of targeted radionuclide therapy can offer the cells the opportunity to repair some of their sublethal lesions (64–67). Nuclear DNA plays a central role in response to targeted radionuclide therapy, but other cellular sub-compartments including the mitochondria and cell membrane might also be strongly involved in situations of heterogeneous energy deposits (68–74). Therefore, the biology of the irradiated tissue and its interaction with its environment might play an even more pronounced role in targeted radionuclide therapy than EBRT, and bystander and abscopal effects involving activation of signaling pathways and the immune system should probably be investigated more accurately (75–77). The consequences are that the absorbed dose-effect might be more difficult to establish and radiation-induced biological effects might be observed in tissues far beyond the physical path length of the α-particles.

## Pre-Targeted α Therapy

All targeted therapies rely on the ability of the vector to find its target and to allow the associated cytotoxic agent to deliver the cell-killing effect. Advances in genetic engineering have led to the development of many molecules that can be radiolabeled and used for RIT. However, despite the growing number of designed antibody fragments and fusion proteins, treatments are often hampered by less than optimal pharmacokinetics. The key lies in finding a balance between tumor radiation uptake and removal of circulating radioactivity. Rapid clearance of unbound radioimmunoconjugates is essential for limiting the absorbed dose to normal organs, but a too short a retention time in blood will result in a too short targeting time, and thus in the delivery of a too low absorbed dose to malignant cells.

This pharmacokinetic challenge can be handled by separating physically and temporally the targeting phase from the delivery of the ionizing radiation, an approach generally referred to as pre-targeted radioimmunotherapy (PRIT) (78, 79). A number of PRIT regimens, all based on the same essential principle, have been proposed since the pre-targeting concept was proposed by Goodwin et al. in 1988 (80). In the first step, a targeting immunoconjugate (pre-targeting molecule) is administered and sufficient time is allowed for its localization at tumor-associated antigen sites. As the pre-targeting molecule does not carry any cytotoxic substance, normal tissues are not affected by lengthy circulation times during the distribution phase. Then, unbound immunoconjugate molecules can be removed from the circulation using a clearing agent, before injecting the radiolabeled vector (effector molecule). The effector molecule is a small molecule designed to rapidly diffuse into tumors and cancer cell clusters, where it will specifically bind to the antigen-associated pre-targeting molecules. The fast clearance of unbound effector molecules improves the tumor-to-normal tissue ratios of absorbed dose compared with directly labeled immunoconjugates. With pre-targeting, no trade-off needs to be made between efficient targeting/penetration/tumor residence time and protection of dose-limiting normal tissues.

Efficient interaction between the pre-targeting molecule and the effector molecule has been achieved using a handful of techniques, particularly those based on streptavidin-biotin (81) or bispecific antibodies (82). Of the radionuclides with potential use in TAT, some appear more suitable than others when factors such as availability and daughter nuclides are taken into account, in addition to chelation and conjugation chemistry. In particular, two promising candidates for efficient therapy emerge: ^211^At and ^213^Bi. However, they both have short *T*_1/2_ (7.2 h and 45.6 min, respectively), which put high demands on the distribution of radiolabeled vectors to ensure favorable absorbed dose ratios. This issue could be overcome by using a pre-targeting strategy, thereby increasing the therapeutic potential of these short-lived α-particle emitters.

Several preclinical studies have shown the benefits of pre-targeted α therapy (PTAT), mainly in hematological cancers, such as AML (83), non-Hodgkin lymphoma (84), anaplastic large cell lymphoma (85), and adult T-cell leukemia (85). PTAT for disseminated ovarian carcinoma was evaluated in one study in which ^211^At-PRIT (1.5 MBq) and ^211^At-RIT (0.9 MBq) were compared in a mouse model of i.p. TAT (86). The administered activities were based on the previously estimated MTAs for the two regimens and resulted in equal tumor-free fractions (TFF; 0.45) 8 weeks after irradiation; however, the mice treated with ^211^At-PRIT had smaller tumors and lower ascites incidence. This indicates that pre-targeting can improve the outcome also of i.p. TAT, although the greatest gain of PTAT is generally considered to be in systemic treatments.

## Summary and Future Perspectives

Radioimmunotherapy with short-ranged, high-efficiency α-particles is a very attractive and promising treatment strategy. α-Particles have an advantage in targeted therapy because of their exceptionally high cell-killing ability. Therefore, different from RIT with β^–^-particles, α-particle emitters labeled to a targeting vector can directly kill single cancer cells (by self-irradiation). Several completed or on-going clinical trials using TAT have shown its feasibility for treating disseminated and/or micro-metastatic malignancies without significant or insurmountable problems of toxicity. Although the definition of micrometastases is vague, in clinical oncology occult metastases (i.e., not detected by routinely used imaging procedures) might involve single tumor cells up to clusters of billions of cells. Therefore, a cocktail of both α- and β^–^-emitting radioconjugates might be more effective in some cases.

The possibility of TAT as a potential curative treatment includes its use as a local boost after initial treatment (e.g., i.p. in EOC), or perhaps as i.v. systemic adjuvant treatment, both targeting micro-metastatic disease. A systemic approach may indeed be of particular interest in patients with EOC that includes retroperitoneal vascularized metastases, e.g., in the lymph nodes. Fractionated RIT is another potentially interesting regimen to improve the therapeutic index, thus resulting in reduced normal organ toxicity while maintaining the therapeutic efficacy (87). Radionuclides that emit Auger electrons could offer an alternative approach compared with the nuclides described in this article, reviewed elsewhere (88). Auger electrons are energetically very weak (< <1 keV) and have a path length in tissue that is far shorter than that of α-particles. However, to effectively damage DNA molecules, the Auger emitter has to bind to the DNA.

The therapeutic outcome of TAT is influenced by a number of crucial issues that all need to be handled, e.g., the specificity of the antibody/targeting construct; the level of antigenic expression on the tumor cells; the potential loss of immunoreactivity of the antibody/targeting construct; the amount of unlabeled antibody/targeting construct after injection; the existence of diffusion barriers that hinder the penetration of the antibody/targeting construct into the tumors; the choice of radionuclide (half-life and path length); too low specific radioactivity; and for the i.p. situation, any extra peritoneal location of tumor cells.

A major issue that may hamper wide implementation in the clinic and that needs to be simultaneously addressed is the availability of suitable α-particle emitters at a reasonable cost (43, 89). Otherwise, TAT will remain just a potentially effective treatment, or a very rarely implemented option. Finally, after safety issues and pharmacokinetics have been established, for all types of malignancies that might benefit from TAT/PTAT, we need to conduct randomized, controlled, clinical studies. These need to include a high enough number of patients to allow meaningful comparison and evaluation of different treatment strategies.

## Conflict of Interest Statement

The authors declare that the research was conducted in the absence of any commercial or financial relationships that could be construed as a potential conflict of interest.
